# Cementless long-stem fixation in periprosthetic clamshell fracture: a biomechanical investigation

**DOI:** 10.3389/fbioe.2025.1604441

**Published:** 2025-05-22

**Authors:** Lingqi Zhu, Li Xie, Mingchang He, Jianyun Miao, Liang Zhou, Lianshui Huang, Hui Liu, Wei Xie, Wenliang Zhai

**Affiliations:** Department of Orthopaedics, The 909th Hospital, School of Medicine, Xiamen University, Zhangzhou, Fujian, China

**Keywords:** periprosthetic clamshell fracture, cementless long-stem, revision hip arthroplasty, Biomechanics, periprosthetic femoral fractures

## Abstract

**Objective:**

To investigate the biomechanical strength of cementless long-stem fixation for the treatment of periprosthetic clamshell fracture.

**Methods:**

Eighteen Sawbones artificial femur models were used and divided into three groups. Group A had an intact medial wall. Group B, the proximal periprosthetic femoral medial wall was osteotomized to simulate periprosthetic clamshell fractures. Group C, following identical osteotomy to Group B, cerclage wiring was applied to fix the medial wall fracture fragment. After molding, the cementless long-stem were implanted in all models, which were then evaluated through axial compression tests, torsion tests, and axial failure tests. The axial stiffness, axial displacement, torsional stiffness, torque, and maximum failure load were recorded and statistically analyzed.

**Results:**

In the axial compression tests, no statistically significant differences were observed among Groups A, B, and C in terms of axial displacement, axial stiffness, or maximum failure load (the displacement greater than 3 mm). However, in the torsional tests, Group B showed significantly lower torsional stiffness or torque compared to Groups A and C (*p* < 0.05). No significant differences in torsional stiffness or torque were detected between Groups A and C.

**Conclusion:**

The integrity of the femoral medial wall is crucial for femoral stem stability. In case of medial wall fracture, while cementless long-stem implantation can achieve satisfactory axial stability, its torsional stability remains inadequate. The application of supplemental cerclage wiring for medial wall fragment fixation provides reliable improvement in torsional stability of the stem.

## Introduction

The proximal periprosthetic femoral fractures (PFFs) occurring during or after total hip arthroplasty (THA) has been increasing annually, which severely impacts the quality of life and lifespan of patients (Miettinen et al., 2023). When PFFs lead to stem instability, revision surgery is often required to restore stability and functionality to the hip joint (Gausden et al., 2024). Clinically, X-ray or CT imaging is primarily used to classify PFFs according to the Vancouver classification, thereby distinguishing between stable and unstable prostheses (Brady et al., 2000). The Vancouver classification system is divided into three types: A, B, and C. It categorizes fractures based on the location of the fracture, the stability of the prosthesis, and residual proximal femoral bone stock, and it demonstrates excellent repeatability and simplicity to guide the treatment. Type A fractures occur at the greater or lesser trochanters of the femur and do not affect the stability of the prosthesis. They are divided into two subtypes: AG fractures involve the greater trochanter, while AL fractures involve the lesser trochanter. Type B fractures are located at the prosthesis stem and can be further divided into three subtypes: B1, where the fracture occurs at the prosthesis stem without instability; B2, where the fracture occurs at the prosthesis stem with instability; and B3, where the fracture occurs at the prosthesis stem with instability and poor bone stock. Type C fractures are located below the tip of the prosthesis stem with stability (Duncan and Masri, 1995).

The medial calcar is located deep within the lesser trochanter of the femur, extending longitudinally from the posterior aspect of the femoral neck to the posterior aspect of the lesser trochanter as a dense bony plate. It is an important weight-bearing structure in the upper femur and plays a significant role in weight-bearing, internal fixation of fractures, and hip arthroplasty. Fractures of the medial wall involving the medial calcar can influence the biomechanical properties of the proximal femur, potentially leading to instability (Li et al., 2020). Therefore, reduction and fixation of the medial wall are particularly crucial. The medial femoral wall fractures were initially described as an intraoperative fracture in 1989 (Mallory et al., 1989). However, in the original Vancouver classification, only lesser trochanter fractures (Vancouver ATL type) were categorized, with no specific distinction made for periprosthetic femoral medial wall fractures (Duncan and Masri, 1995). Despite the similarities between ATL type fractures and medial wall fractures, ATL type fractures do not affect the stability of the medial wall. More recently, in 2011, Van Houwelingen and Duncan (Van Houwelingen and Duncan, 2011) termed it a pseudo ATL or New B2 fracture to distinguish it from the ATL type fracture as described in the original Vancouver classification. They noted that risk factors for this fracture pattern include the use of tapered, proximally coated, cementless stems in primary THAs. In 2014, Capello et al. (Capello et al., 2014) defined the “clamshell fracture” and further subdivided the pseudo ATL or New B2 fracture into A1 and A2 subtypes within the Vancouver classification. When a periprosthetic fracture involves the medial wall with a stable stem, it is classified as subtype A1, whereas if there is stem instability, it is classified as subtype A2.

The distinction between a stable and unstable femoral stem is particularly crucial for accurate classification, as it influences the selection of treatment strategies. However, the clinical assessment of stem instability typically relies on a combination of patient clinical presentation and radiographic results (Kiernan et al., 2013). When there is clear evidence of stem subsidence, tilt, or rotation on imaging results, diagnosing stem instability is not challenging (Marshall et al., 2017). However, when a periprosthetic fracture does not present with obvious radiographic signs of instability, determining its classification can be quite difficult. Among patients with type B1 fractures, those who underwent open reduction and internal fixation (ORIF) experienced a notably higher failure rate. In the revision group, failure occurred in 17.1% of cases. The failure rate for revision combined with ORIF was 18.7%, for plate fixation alone it was 33.9%, and for cerclage fixation it reached 43.9%. This discrepancy may be attributed to the clinical misclassification of some B2 fractures as B1, leading to treatment with internal fixation instead of revision surgery (Lindahl et al., 2006).

Some researchers have utilized the calculation of the prosthesis remaining attachment index (RAI) during X-ray examination to assess prosthesis stem stability. When the RAI was greater than 2/3, the prosthesis was considered stable, corresponding to a B1 fracture. If the RAI was less than 2/3, it was classified as a B2 fracture, indicating loss of prosthesis stability, and it was found that this method was very similar to CT scan results in determining prosthesis loosening (Andriamananaivo et al., 2020). RAI was measured as A/B ratio on Anterior-Posterior (AP) view where A is the minimal remaining anchorage height and B is the total initial height. This method has gained recognition from many researchers (Kastner et al., 2023; Sattar et al., 2023). In patients with the clamshell fracture (Vancouver classification B2 type) and a remaining attachment index of less than 2/3, cementless long-stem treatment is commonly employed (González-Martín et al., 2022; Schöfl et al., 2022; Frangie et al., 2023; Siljander et al., 2024). However, there is a lack of biomechanical evidence regarding its sufficient initial stability. At the same time, for patients with periprosthetic clamshell fractures in clinical practice, whether to perform reduction and fixation of the medial wall fracture fragment during femoral stem revision surgery remains controversial. To address this, we designed biomechanical experiments to investigate the biomechanical strength of cementless long-stem and cerclage wiring fixation in the treatment of periprosthetic clamshell fractures, aiming to provide guidance for clinical surgical decision-making.

## Materials and methods

### Specimens and study groups

**FIGURE 1:**
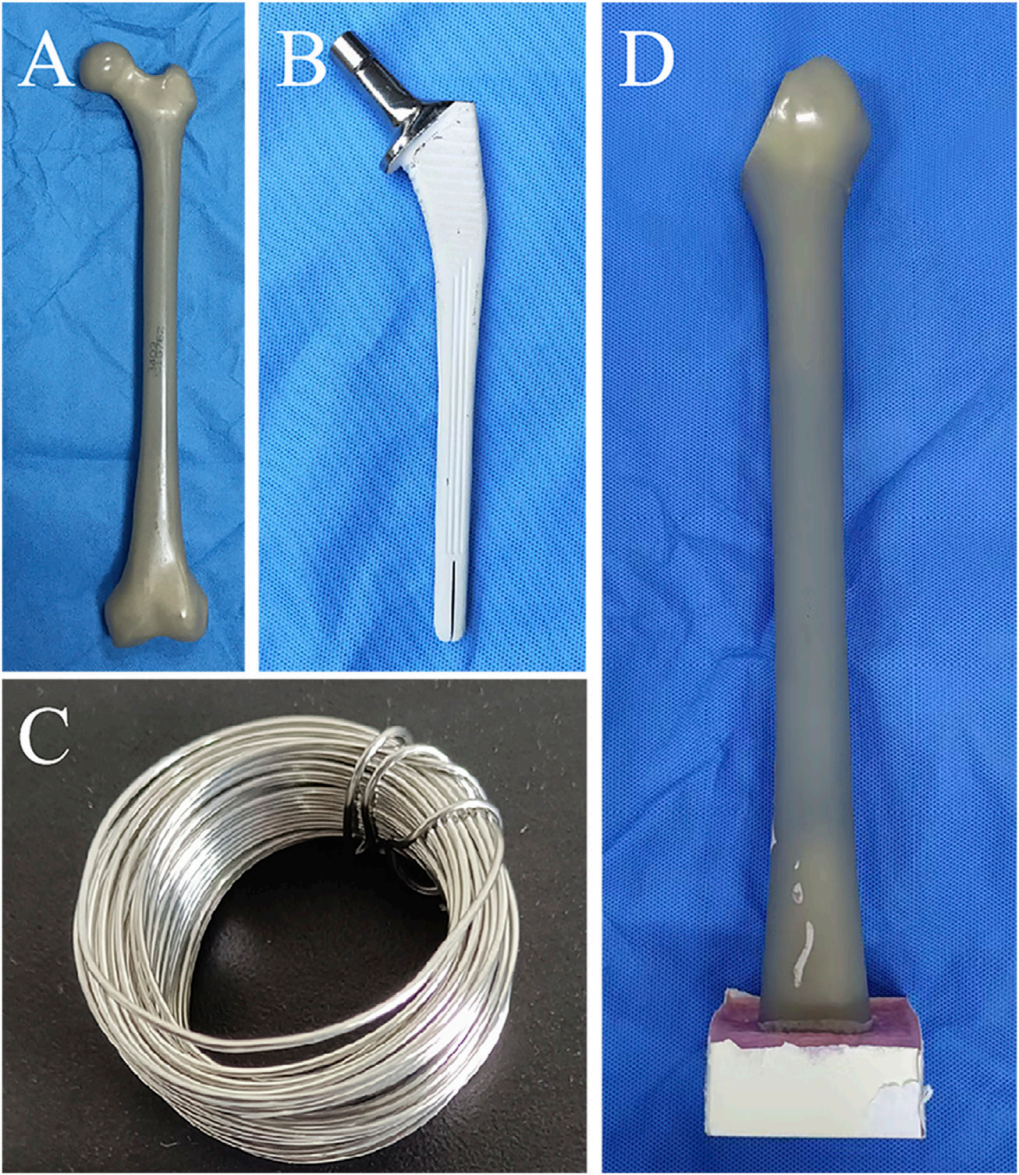
**(A)** The artificial femur model. **(B)** The cementless long-stem (Corail revision stem, Hydroxyapatite plasma-coated press fit, Johnson, United States). **(C)** Wire. **(D)** The artificial bone model after femoral neck osteotomy.

**FIGURE 2:**
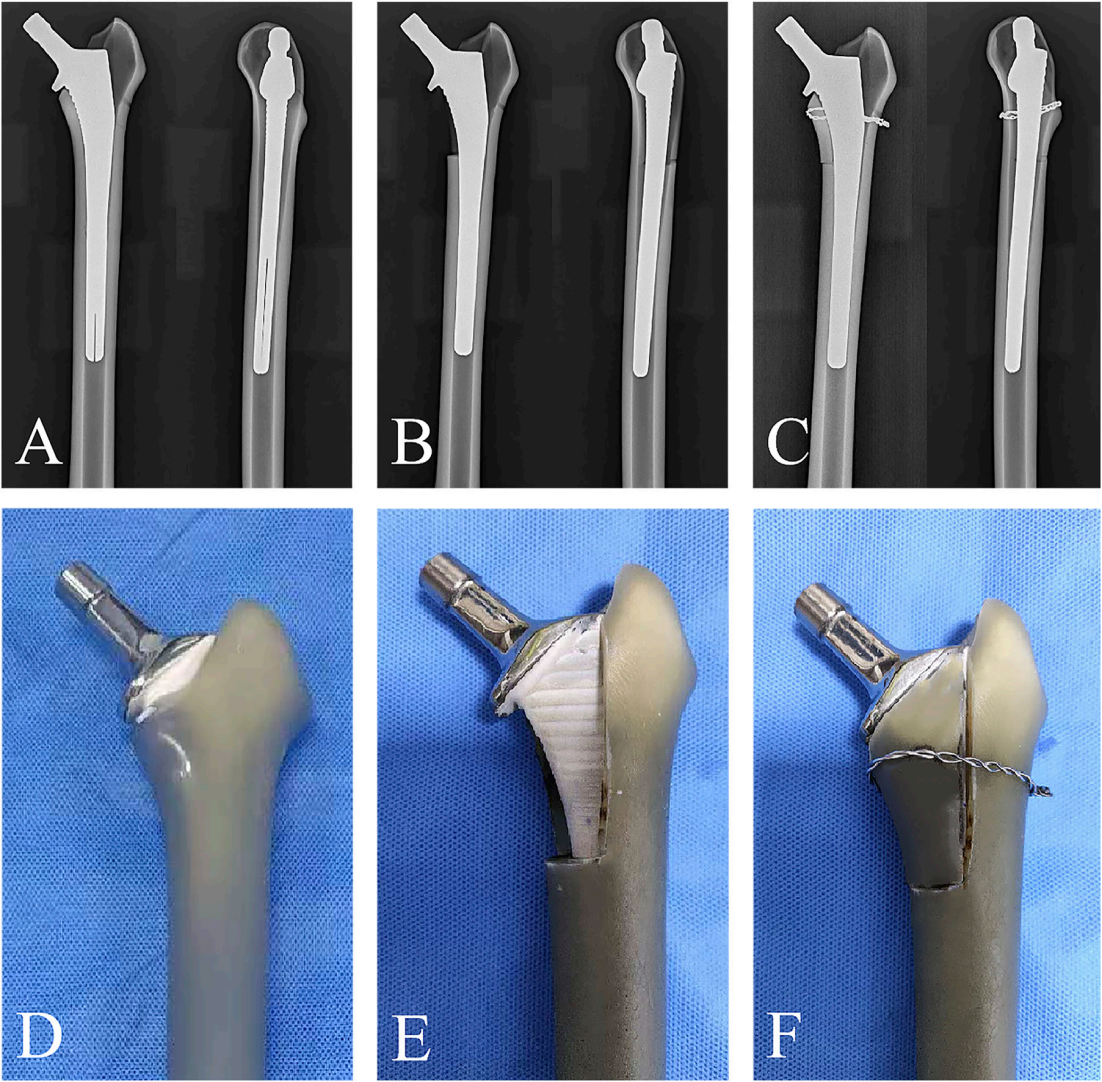
**(A–C)** The radiographic images that describing the fixation strategies of Groups A, Group B, and Group C. **(D–F)** The photograph images that describing the fixation strategies of Groups A, Group B, and Group C.

Eighteen artificial femur models (left femur model 3,403; Sawbones, Pacific Research Laboratories, Vashon, WA, United States) (Figure 1A) were randomly divided into three groups. Firstly, the femoral neck of all artificial bones were resected using an oscillating saw according to standard osteotomy techniques (Figure 1D), and then, the primary femoral stem (Corail stem, Hydroxyapatite plasma-coated press fit stem, Johnson, United States) was installed using the standard femoral stem installation method. A 2:3 ratio of fracture length to stem length (RAI) was applied as the stem stability cutoff to differentiate Vancouver B1 from B2 fractures (Andriamananaivo et al., 2020). So, we defined the fracture line length as 40% of the primary femoral stem length to establish the unstable medial fracture model and removed the selected fracture region that contained the entire lesser trochanter and the portion of the surrounding medial cortical wall (Kastner et al., 2023). To simulate different treatment plans for medial wall fractures, we divided the model into three groups. Group A: intact medial wall group. This means that in this group, the models had an intact medial wall without any fractures, and the medial wall structure remains whole, followed by the implantation of a cementless long-stem. This group can serve as a control group to be compared with other groups that had medial wall fractures. Group B: the medial wall fracture group, which used a fracture model constructed based on previous literature reports. The length of the fracture line was 40% of the primary stem length, followed by the implantation of a cementless long-stem, without fixation of the medial wall fracture fragment. Group C: the medial wall fracture group, had a fracture line length of 40% of the primary stem length, followed by cementless long-stem implantation with supplemental a double-bundle cerclage wiring (diameter 1mm, Jiangsu Aidier Company, China) fixation of the upper part of the lesser trochanter (Figure 1C). Subsequently, the insertion of a cementless long-stem (Corail revision stem, Hydroxyapatite plasma-coated press fit, Johnson, United States) (Figure 1B) was inserted into all models after the creation of clamshell fractures to simulate the surgical treatment of a periprosthetic fracture (Figures 2D–F). To avoid mismatched implantation, all procedures were performed by the same experienced associate chief physician who was familiar with the stem system and performed the standardized implantation according to the manufacturer’s recommendations. After the model construction and stem installation were completed, X-ray examination was performed on all models to ensure all stems were located the correct position (Figures 2A–C). Subsequently, the specimens were osteotomized at the distal diaphysis and fixed on the biomechanical testing machine platform with polymethyl methacrylate (PMMA) for testing.

### Biomechanical testing

**FIGURE 3:**
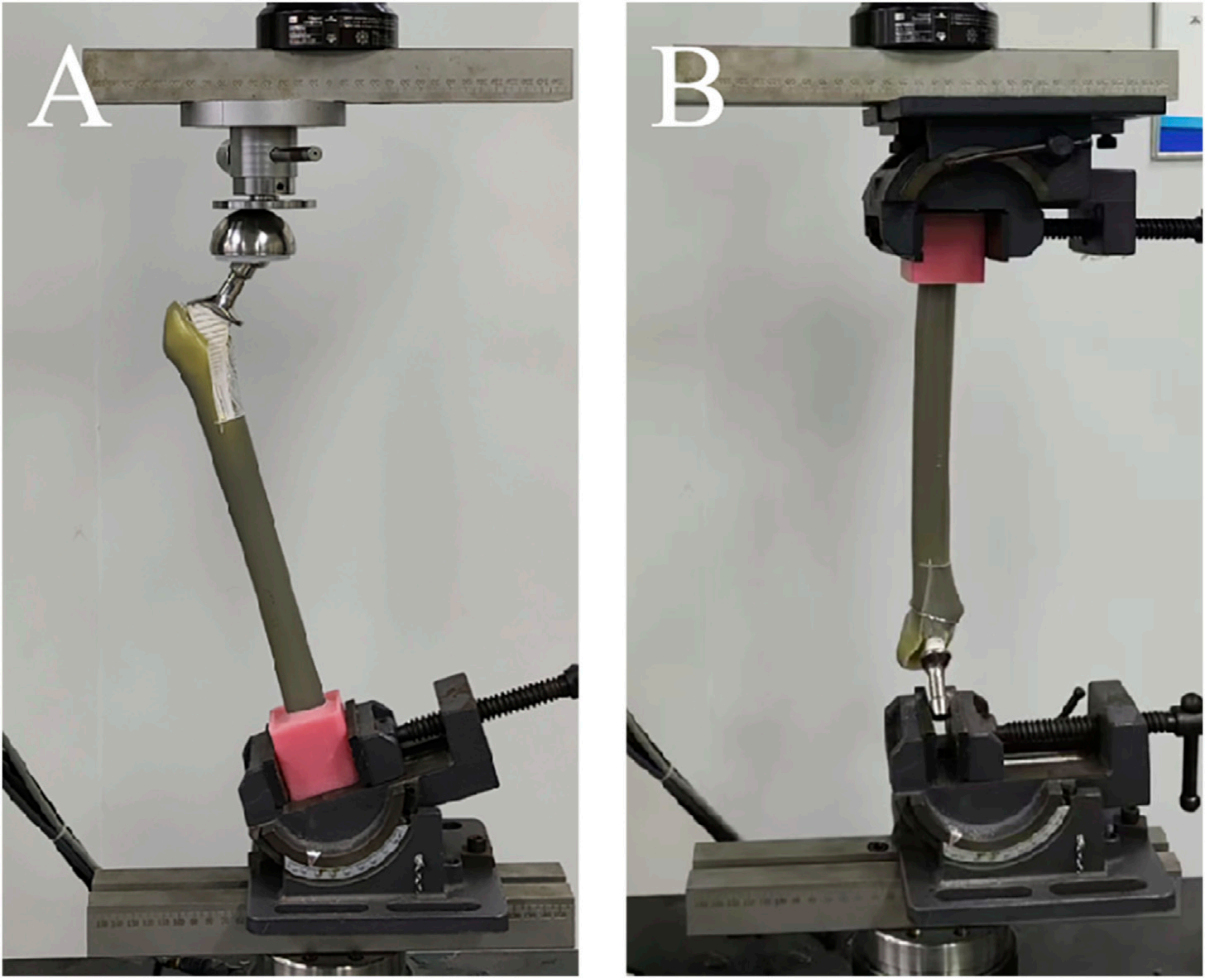
**(A)** The picture of artificial femur model in axial compression test. **(B)** The picture of artificial femur model in torsion test.

All models were tested by the mechanical testing machine (MTS Bionix Servo-hydraulics Test Systems Model 370.02; MTS Systems, Eden Prairie, MN, United States) at Xiamen Medical Device Research and Development Testing Center. To simulate the force-bearing state of a normal adult femur during weight-bearing activities, the model was fixed on the testing machine at an adduction of 7°, ensuring that the axial load passed through the mechanical axis of the artificial femur (Wähnert et al., 2020). Before conducting official biomechanical tests, we applied an axial compression load of 0–350 N to the specimens for 30 s, repeated three times consecutively, to ensure a tighter connection between the stem and the specimen, thereby reducing the potential impact of specimen relaxation and creep on the test results. This method had already been applied in our previous studies (Xie et al., 2024a; Xie et al., 2024b). The single-cycle axial compression load was increased from 0 N to 2100 N at a rate of 3 mm per minute (Figure 3A). According to a previous test protocol, adequate primary stability can be assumed if the model can withstand a defined maximum load of 250% of body weight. Therefore, we chose 2100 N (three times the body weight of an adult who is 70 kg) as the maximum axial load. The axial compression displacement (mm) was recorded at the pressures at 700 N, 1400 N and 2100 N, and the stiffness (N/mm) of axial compression was calculated as the load (N) divided by the displacement (mm). In the torsion test (Figure 3B), the direction of torsion was the external rotation of the proximal femur, the torsion angle was loaded from 0° to 5°at a rate of 0.05°/s, and the angle and corresponding torque were recorded. The torsional stiffness (Nm/°) was calculated by dividing the applied torque (Nm) by the angle of rotation (°). After completing the axial compression and torsion experiments, for all specimens, an axial compressive load was continuously applied incrementally from 0 N at a rate of 10 N/s until the specimens failure. Then, the maximum load was recorded. During the application of axial compression load, stem loosening was defined as a stem displacement greater than 1 mm, while the failure of stem-bone-structure complex was defined as a stem displacement greater than 3 mm or a rapid drop in the load-displacement curve (Kastner et al., 2023).

### Statistical analysis

The SPSS software (V26; IBM SPSS^®^ Statistics) was used for statistical analysis following the completion of the tests. Axial compression loads with corresponding displacements, along with torsional torque and their angular rotations, were directly recorded by the testing machine’s sensors. The Shapiro-Wilk test was used to check normality for continuous variables. Normally distributed continuous variables were described as mean ± standard deviation. One-way analysis of variance was performed to compare the differences in compression stiffness, compression displacement, torsion stiffness, torque, ultimate compression load. *p* < 0.05 was considered statistically significant.

## Results

In all the groups, no stem loosening or fracture was observed in each group of specimens before the implementation of the destruction experiment.

**FIGURE 4:**
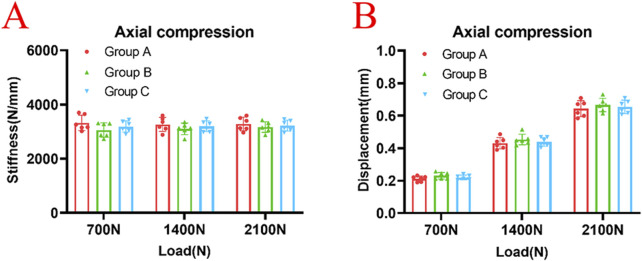
There were no statistically significant differences in axial stiffness **(A)** or displacement **(B)** among Group A, Group B, and Group C under the axial loads of 700N, 1400N and 2100N (*p* > 0.05).

In the axial compression test, the average stiffnesses of the Group A under axial loads of 700 N, 1400 N, and 2100 N were 3,324 ± 282 N/mm, 3,265 ± 258 N/mm, and 3,282 ± 250 N/mm, respectively. The average stiffnesses of the Group B were 3,057 ± 266 N/mm, 3,102 ± 212 N/mm and 3,168 ± 193 N/mm, respectively. The average stiffnesses of the Group C were 3,190 ± 206 N/mm, 3,199 ± 199 N/mm, and 3,221 ± 201 N/mm, respectively. There were no statistically significant differences in average stiffness among Groups A, B, and C (*p* > 0.05) (Figure 4A). The average displacements of the Group A under axial loads of 700 N, 1400 N, and 2100 N were 0.21 ± 0.174 mm, 0.43 ± 0.349 mm, and 0.64 ± 0.492 mm, respectively. The average displacements of the Group B were 0.23 ± 0.203 mm, 0.45 ± 0.331 mm and 0.67 ± 0.411 mm, respectively. The average displacements of the Group C were 0.22 ± 0.144 mm, 0.44 ± 0.272 mm, and 0.65 ± 0.410 mm, respectively. There were no statistically significant differences in average displacements among Groups A, B, and C (*p* > 0.05) (Figure 4B).

**FIGURE 5:**
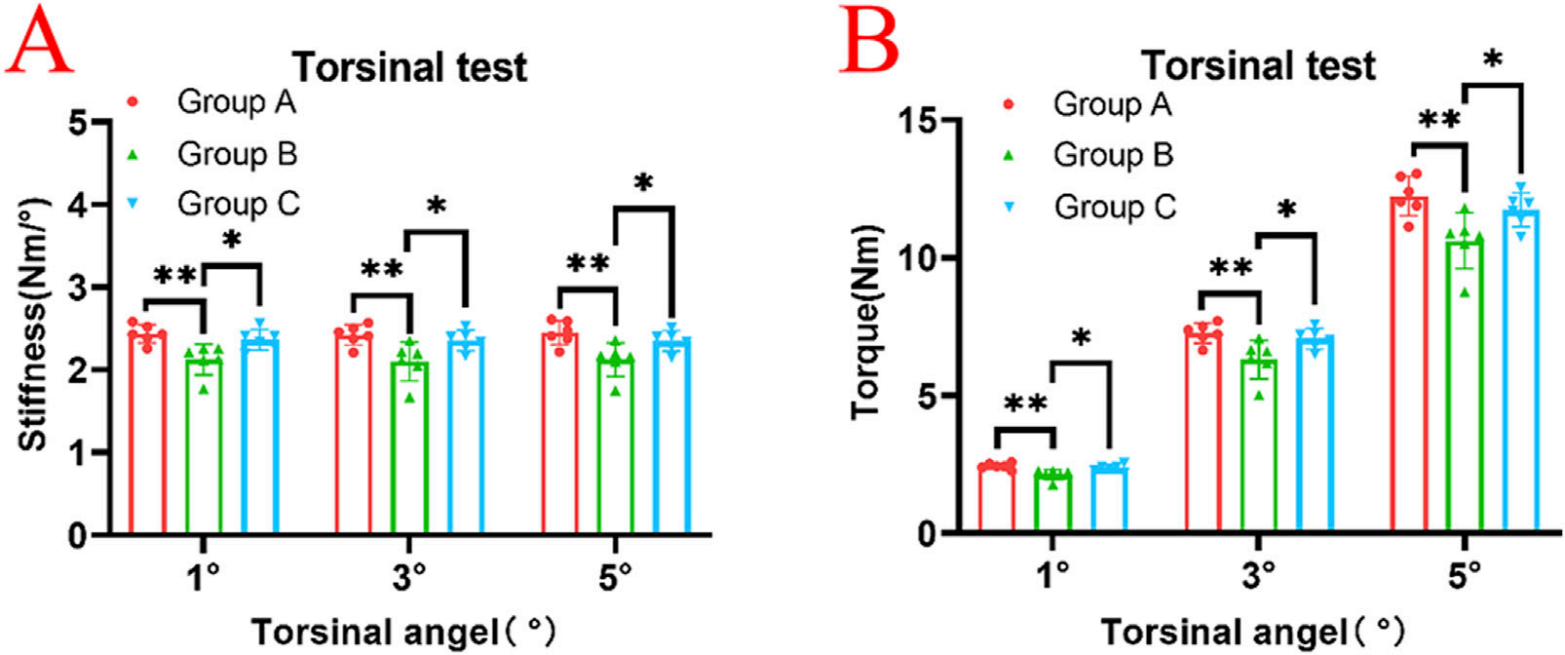
**(A)** In the torsion test, at torsional angles of 1°, 3°, and 5°, Group B exhibited significantly lower torsional stiffness compared to both Group A and Group C (*p* < 0.05), while no statistically significant difference was observed between Group A and Group C (*p* > 0.05). **(B)** In the torsion test, at torsional angles of 1°, 3°, and 5°, Group B exhibited significantly lower torque values compared to both Group A and Group C (*p* < 0.05), while no statistically significant difference was observed between Group A and Group C (*p* > 0.05).

In the torsion test, the average torsional stiffness of Group A at degree of 1°, 3° and 5° were 2.44 ± 0.110 Nm/°, 2.42 ± 0.124 Nm/°, 2.45 ± 0.145 Nm/°, respectively. The average torsional stiffness of Group B were 2.13 ± 0.184 Nm/°, 2.10 ± 0.234 Nm/°, 2.12 ± 0.203 Nm/°, respectively. The average torsional stiffness of Group C were 2.37 ± 0.127 Nm/°, 2.36 ± 0.125 Nm/°, 2.35 ± 0.124 Nm/°, respectively. The Group B had lower torsional stiffness than Group A and Group C (*p* < 0.05) (Figure 5A). The average torque of Group A at degree of 1°, 3° and 5°, were 2.44 ± 0.110 Nm, 7.26 ± 0.365 Nm, and 12.24 ± 0.721 Nm, respectively. The average torque of the Group B were 2.13 ± 0.184 Nm, 6.30 ± 0.703 Nm, and 10.61 ± 1.009 Nm, respectively. The average torque of the Group C were 2.37 ± 0.127 Nm, 7.06 ± 0.377 Nm, and 11.74 ± 0.615 Nm, respectively. The torque of Group B was significantly lower than that of Group A and C (*p* < 0.05) (Figure 5B). There were no statistically significant differences between Groups A and C in terms of torsional stiffness and torque (*p* > 0.05).

**FIGURE 6:**
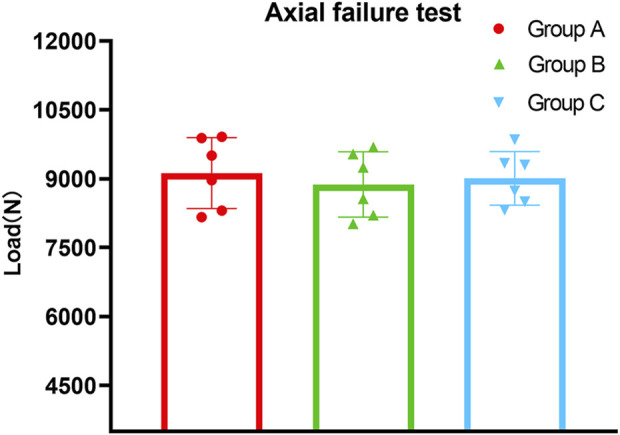
The results of axial failure test showed that the ultimate load (displacement greater than 3 mm) of Group B was lower than that of Group A and Group C, while no statistically significant difference was observed between Group A, Group B and Group C (*p* > 0.05).

In the axial compression failure test, the average ultimate load (displacement greater than 3 mm) of Group A was 9,123 ± 770 N, Group B was 8,876 ± 711 N, and Group C was 9,009 ± 584 N. There were no statistically significant differences in average ultimate load among Groups A, B, and C (*p* > 0.05) (Figure 6).

## Discussion

Hip arthroplasty is an effective treatment for symptomatic end-stage hip osteoarthritis and femoral neck fracture (Patel et al., 2023; Xian et al., 2024). With the aging of the population and the increasing demand for quality of life, the number of hip arthroplasty is expected to grow further (Agni et al., 2023). PFFs are a serious complication of joint replacement surgery, accounting for approximately 3.5% of hip revision surgeries, and this proportion is expected to increase in the future (Schwartz et al., 2020). They add to the economic burden on patients and society and are associated with a high mortality rate (Miettinen et al., 2023). Revision surgery is often required when these fractures predominantly affect the medial proximal femoral structures, which may compromise the stability of the prosthetic stem (Gausden et al., 2024). The lesser trochanter fracture (the Vancouver type ALT fracture) is an uncommon occurrence in PFFs. Since it is an avulsion fracture of the attachment of the iliopsoas, it does not destabilize the stem and can be treated nonsurgically. The “Clamshell-Fracture” pattern, as newly delineated by Capello et al. (Capello et al., 2014), is characterized by a fracture that localizes to the area of the lesser trochanter and extends to include a segment of the proximal medial femoral cortex. It mainly bears compressive stress during weight-bearing movement, and the aim of both intramedullary and extramedullary fixation is to rebuild the intact physiological structure of the proximal femur, thus restoring its mechanical stability. Strauss et al. (Strauss et al., 2007) conducted biomechanical tests using an artificial composite femur and found that the femoral head bore more compressive stress than the femoral neck when the medial cortex of the proximal femur was crushed, which triggered the inversion of the head and neck and led to fracture breakage. The Clamshell-Fracture is usually seen within 6 weeks of the index procedure, typically following insertion of a tapered, cementless stem within a demineralized femur. This may be due to an unrecognized intraoperative fracture that subsequently displaced under load, or it may occur soon after, during rehabilitation. More recently, Van Houwelingen and Duncan (Van Houwelingen and Duncan, 2011) labeled it a pseudo ATL or New B2 fracture so as to not confuse it with an ATL fracture as described in the original Vancouver classification system. It is important to distinguish this fracture from the type ATL, because it is associated with destabilization of the stem and requires early reintervention. Although the decision to perform revision surgery on the prosthetic stem can be based on whether the stem is loose, there is still insufficient clinical and biomechanical evidence regarding the management of the medial fracture fragment, especially when revision with a cementless long-stem is performed.

Some studies have investigated the biomechanical strength of primary stems in medial wall fractures and have found that when the fracture line length of the medial wall exceeds 40% of the primary stem length, it can compromise the initial stability of the stem. In the treatment of unstable PFFs, revision surgery is typically required (Kastner et al., 2023; Sattar et al., 2023). In hip revision surgery, it is crucial to achieve good fixation at the prosthetic stem-bone interface. The use of cementless long stems in revision surgery is becoming increasingly popular, as stability is difficult to achieve with cemented or proximally fixed prostheses, especially in cases of proximal bone loss and osteoporosis. Because the hydroxyapatite porous coating grew inward and adhered to the bone significantly more, it resulted in a more even distribution of bone over the cementless stem’s surface (Coathup et al., 2001). This could imply potential reductions in stress shielding and limitations on osteolysis caused by wear particles. Clinical studies have also confirmed that the hydroxyapatite coating on biological stems can promote osseointegration and facilitate the repair of femoral bone defects, even in cases of significant bone loss in the femur (Reikerås, 2017; Critchley et al., 2020). Achieving sufficient initial stability of the cementless stem is a prerequisite for the ingrowth of hydroxyapatite coating into the bone. Nevertheless, there is a lack of experimental evidence regarding the biomechanical strength of cementless long-stems when occurred medial wall fractures. To address this, our study investigated the initial stability of cementless long-stems in the treatment of clamshell periprosthetic fractures. Previous literature reports that when the length of the fracture line reaches 40% of the stem length, the initial stability of the stem is significantly reduced compared to the normal group, highlighting the importance of the medial wall bone for the stability of the prosthesis stem.

In this experiment, in order to test the biomechanical strength of the prosthesis stem during medial wall fracture, we established a medial wall fracture group (Group B), with a fracture line length of 40% of the primary stem length. Subsequently, we treated it with a cementless long-stem revision. Since the model’s fracture line length was less than 40% of the cementless long-stem length (the cementless long-stem was longer than the primary stem), the RAI after revision treatment was greater than 2:3, thus it is considered that the cementless long-stem has sufficient stability. This was consistent with our axial compression test results, which showed that the axial displacement and stiffness of Group B have only a small difference from the intact medial wall group (Group A) (*p* > 0.05). This demonstrated good initial axial stability of the cementless long stem due to its longer length. From a biomechanical perspective, in patients with these conditions, the goal of minimizing surgical trauma can be met without the need for additional fixation of the medial wall fracture, while still ensuring satisfactory initial stability. In another biomechanical study, the researchers used cerclage wires to fix the lower end of the medial wall fracture, and although the biomechanical strength was still weaker than the normal group, the difference was not statistically significant. However, these findings were limited to axial compression testing. During human activity, the femoral stem prosthesis not only bears axial compressive stress but also torsional stress (Johnson et al., 2020). Therefore, we added a torsional test. The results revealed that, unlike our compression test results, the torsional test found that the torsional stability of Group B was significantly lower than that of Group A. This indicated that for periprosthetic clamshell fracture patients, even with the treatment of cementless long-stem revision, sufficient initial stability cannot be achieved postoperatively. Early ambulation may still lead to early loosening of the prosthesis, affecting the surgical outcome.

Cerclage wire fixation had been demonstrated as an effective treatment for PFFs (Rilby et al., 2022; Yun et al., 2023). Although the European Hip Society’s consensus, based on their findings, advocates revision surgery with cerclage wire fixation as the preferred approach for Vancouver B2 fractures (Thaler et al., 2023), some studies demonstrate that ORIF alone may achieve good fracture healing outcomes (Joestl et al., 2016; Martinov et al., 2022). However, given that PFF patients are predominantly elderly, ORIF still necessitates prolonged postoperative bed rest, potentially leading to immobilization-related complications. In contrast, while revision surgery combined with cerclage fixation does entail increased surgical trauma and risks, it enables earlier postoperative functional rehabilitation and weight-bearing activities - a critical determinant of recovery efficacy in elderly populations. Based on Group B, we applied cerclage wire to fix the medial wall fracture and investigate the significance of this auxiliary wire in restoring the torsional stability of the prosthetic stem. The results indicated that the torsional stability of the cerclage wire group (Group C) was significantly enhanced compared to Group B (*p* < 0.05), and there was no significant difference from Group A (*p* > 0.05), suggesting that the combined cerclage wire fixation can effectively restore the torsional stability of the prosthetic stem. Although some researchers argued that the axial stability of the prosthetic stem was not affected after revision surgery, from a biomechanical perspective, not using the reduction of the medial wall fracture fragment and cerclage wire fixation can reduce surgery time, blood loss during surgery, risk of infection and avoid iatrogenic damage. However, according to our experimental results, the medial wall is crucial for maintaining the torsional stability of the proximal femoral prosthetic stem. When a periprosthetic fracture involves the medial wall, revision surgery using a cementless long stem—without reducing and fixing the medial wall—fails to ensure sufficient initial stability. Consequently, early ambulation may still lead to prosthetic loosening.

Our study has several limitations. Firstly, we used an artificial composite femur model that was not designed to represent osteoporosis, whereas most PFFs with proximal medial wall fractures in clinical settings occur in elderly patients with osteoporosis. This model, therefore, may not accurately simulate the clinical scenario. Secondly, our models lacked soft tissues and ligaments, leading to standardized osteotomies that do not fully reflect the complexity of actual clinical fractures in patients. Thirdly, we used single-cycle axial compression load testing instead of cyclic loading, which does not adequately simulate physiological gait conditions (Li et al., 2024). Meanwhile, in this study, the stability of the model was indirectly assessed through stem displacement measured by machine-embedded sensors; however, the method could not analyze the creep and displacement of the medial fracture fragment. Therefore, while the results of this study indicated that using a cementless long stem with cerclage wire to reconstruct the femoral medial wall provides favorable biomechanical strength and adequate initial stability, additional biomechanical validation and clinical trials remain necessary to determine whether this technique can reliably enhance fixation stability and promote fracture healing in clinical applications.

## Conclusion

The femoral medial wall is crucial for maintaining both the axial and torsional stability of the femoral stem. While cementless long-stem revision demonstrates adequate axial stabilization in periprosthetic clamshell-type fractures, this approach does not effectively restore proximal femoral torsional resistance. Therefore, a combined treatment with cerclage wiring fixation should be employed.

## Data Availability

The original contributions presented in the study are included in the article/supplementary material, further inquiries can be directed to the corresponding authors.
